# Crystal Structure of *Plasmodium knowlesi* Apical Membrane Antigen 1 and Its Complex with an Invasion-Inhibitory Monoclonal Antibody

**DOI:** 10.1371/journal.pone.0123567

**Published:** 2015-04-17

**Authors:** Brigitte Vulliez-Le Normand, Bart W. Faber, Frederick A. Saul, Marjolein van der Eijk, Alan W. Thomas, Balbir Singh, Clemens H. M. Kocken, Graham A. Bentley

**Affiliations:** 1 Institut Pasteur, Unité d’Immunologie Structurale, Département de Biologie Structurale et Chimie, Paris, France; 2 CNRS URA 2185, Paris, France; 3 Department of Parasitology, Biomedical Primate Research Centre, Rijswijk, The Netherlands; 4 Malaria Research Centre, Faculty of Medicine and Health Sciences, Universiti Malaysia Sarawak, Kuching, Sarawak, Malaysia; Centro de Pesquisa Rene Rachou/Fundação Oswaldo Cruz (Fiocruz-Minas), BRAZIL

## Abstract

The malaria parasite *Plasmodium knowlesi*, previously associated only with infection of macaques, is now known to infect humans as well and has become a significant public health problem in Southeast Asia. This species should therefore be targeted in vaccine and therapeutic strategies against human malaria. Apical Membrane Antigen 1 (AMA1), which plays a role in *Plasmodium* merozoite invasion of the erythrocyte, is currently being pursued in human vaccine trials against *P*. *falciparum*. Recent vaccine trials in macaques using the *P*. *knowlesi* orthologue PkAMA1 have shown that it protects against infection by this parasite species and thus should be developed for human vaccination as well. Here, we present the crystal structure of Domains 1 and 2 of the PkAMA1 ectodomain, and of its complex with the invasion-inhibitory monoclonal antibody R31C2. The Domain 2 (D2) loop, which is displaced upon binding the Rhoptry Neck Protein 2 (RON2) receptor, makes significant contacts with the antibody. R31C2 inhibits binding of the Rhoptry Neck Protein 2 (RON2) receptor by steric blocking of the hydrophobic groove and by preventing the displacement of the D2 loop which is essential for exposing the complete binding site on AMA1. R31C2 recognizes a non-polymorphic epitope and should thus be cross-strain reactive. PkAMA1 is much less polymorphic than the *P*. *falciparum* and *P*. *vivax* orthologues. Unlike these two latter species, there are no polymorphic sites close to the RON2-binding site of PkAMA1, suggesting that *P*. *knowlesi* has not developed a mechanism of immune escape from the host’s humoral response to AMA1.

## Introduction

Human malaria was long thought to be restricted to infection by four *Plasmodium* species: *P*. *falciparum*, *P*. *vivax*, *P*. *malariae* and *P*. *ovale*. It has now been confirmed, however, that natural human infection also occurs with *P*. *knowlesi* [1], a species hitherto associated only with macaque hosts. Human infection by *P*. *knowlesi*, previously confused with infection by the less virulent *P*. *malariae*, is quite widespread in Southeast Asia and can lead to mortality [2–4]. Accordingly, there is a need to include *P*. *knowlesi* in therapeutic and vaccine strategies against human malaria.

Apical Membrane Antigen 1 (AMA1), a type 1 transmembrane protein of the *Plasmodium* parasite, includes an ectodomain, a transmembrane region and a cytoplasmic domain. The ectodomain comprises three domains referred to as Domain 1, Domain 2 and Domain 3. AMA1 is produced in the microneme organelles and transferred to the parasite surface just prior to or during red blood cell (RBC) invasion [5]. First detected in *P*. *knowlesi* [6], AMA1 was later found in other *Plasmodium* species, as well as in other members of the *Apicomplexa* phylum [7–9]. AMA1 appears to be essential for invasion since, for several *Plasmodium* species, antibodies raised against the ectoplasmic region of the protein have been shown to inhibit invasion, and immunization with AMA1 in animal models protects against infection [10–14]. In spite of significant polymorphism, it is a leading malaria vaccine candidate and vaccine formulations based on the *P*. *falciparum* AMA1 ectodomain are currently being pursued in clinical trials [15, 16].

Crystal structures of AMA1 from *Plasmodium* species and other members of the *Apicomplexa* phylum (*P*. *vivax* [17], *P*. *falciparum* [18], *Toxoplasma gondii* [19], *Babesia babesia* [20] and *Neospora caninum* [20]) have revealed the presence of a hydrophobic groove on Domain 1 of the protein. This region is targeted by invasion-inhibitory monoclonal antibodies [21, 22], suggesting that it forms a receptor-binding site. The receptor for AMA1 is the Rhoptry Neck Protein (RON) complex, which is transferred from the rhoptries to the host cell membrane during invasion [23, 24]. In particular, it has been shown in *T*. *gondii* and *P*. *falciparum* that AMA1 interacts directly with the component RON2 of the receptor [25,26]. Furthermore, crystal structures of the complex formed between TgAMA1 or PfAMA1 and a peptide derived from the extracellular region of RON2 from each of these respective species have confirmed that the hydrophobic groove on AMA1 contributes to the receptor-binding site [27, 28]. Moreover, these studies showed that, in addition to the hydrophobic groove, an adjacent surface that becomes exposed upon displacement of a flexible region known as the Domain 2 (D2 loop) also contributes to the RON2-binding site. The AMA1-RON interaction appears to take place at the tight junction, which forms between the merozoite and RBC membranes as the parasite enters the host cell and is a critical component in the invasion process [29]. This model has been subject to controversy, however, with arguments for and against [30–33], showing that further experimental analysis is required to clarify this issue.

The monoclonal antibody (mAb) R31C2, raised in rats against the W1 variant of *P*. *knowlesi* merozoites, is specific for *Plasmodium knowlesi* AMA1 (PkAMA1) and inhibits *in vitro* multiplication of the parasite [6]. R31C2 was the first anti-AMA1 mAb to be characterized (along with mAb R32C3) and has proved to be a useful tool in dissecting the role of AMA1 in RBC infection. Since its Fab fragment is also a highly effective inhibitor, it was concluded that the mAb acts by blocking a receptor-binding site on PkAMA1 [34]. Electron microscopy studies of *P*. *knowlesi* merozoites in the presence of R31C2 have shown that the parasite makes extensive contacts with the RBC surface, characteristic of the random attachment that occurs during the first stage of invasion [35]. However, no apical attachment to the RBC surface nor the subsequent formation of a tight junction between the merozoite and RBC membranes—the ensuing steps in invasion—were observed. Thus AMA1 comes into play downstream from the initial, reversible attachment of the merozoite to the RBC, consistent with the currently accepted model where the AMA1-RON complex is a key component of the tight junction [24, 36–38].

We have recently tested PkAMA1 as a vaccine in monkeys [39]. These experiments showed that repeated immunization with PkAMA1 controlled parasitemia in five out of six monkeys, with the sixth monkey showing a significant delay in the onset of the parasitemia. On the basis of these data, we believe that PkAMA1 is a good vaccine candidate for *P*. *knowlesi* that could be specifically used in Southeast Asia. Here we report the crystal structure of Domains 1 and 2 of the ectoplasmic region of PkAMA1 and of its complex with the Fab fragment of R31C2 which shows that the mAb binds to the hydrophobic groove. In addition, the D2 loop (residues Pro295 to Ser332), which is displaced upon binding the RON2 receptor, remains fixed in the same conformation as the unbound PkAMA1 even though the antibody makes a significant number of direct contacts with this region of the antigen. We also examine the polymorphism of PkAMA1 in the light of these structural data.

## Methods and Materials

### Cloning and expression of PkAMA1

The recombinant PkAMA1 construct comprises residues Pro43 to Pro387 from the PkAMA1 sequence of the *P*. *knowlesi* H strain (Domains 1 and 2; residue numbering from the first residue of the signal sequence, GeneBank accession no. XM_002259303) and an additional 23 C-terminal residues that include the c-myc tag and a hexa-His tag. The synthetic gene was adapted to *P*. *pastoris* codon usage and four potential N-glycosylation sites were mutated as follows: Asn107Lys, Ser178Asn, Asn189Glu and Ser240Arg. (Residues Lys107 and Arg240, Asn178, and Glu189 are present in the *P*. *chabaudi*, *P*. *falciparum* and *P*. *vivax* sequences, respectively). The recombinant gene was maintained in frame with the c-myc-hexahistidine tag by the addition of two bases to the restriction site (XbaI); the sequence at the end of the gene sequence thus codes for an additional glycine [39].

The culture protocol was similar to that reported for the *P*. *vivax* AMA1 construction [40], except for the addition of AEBSF (a serine protease inhibitor, 4-(2-Aminoethyl)benzenesulfonylfluoride hydrochloride) during the induction with methanol in BMMY’ at 0.1 mM. A protease inhibitor was also present (0.4 mM) after recovery of the supernatant. The protein was purified by a metallo-affinity procedure on a ProBond resin and eluted with 20 mM sodium phosphate at pH 6.0, 500 mM NaCl and 500 mM imidazole after prior washing with the same buffer containing 25 mM imidazole to remove contaminating proteins. Crystals were grown by vapour diffusion using the hanging-drop technique at 291 K. The antigen alone was crystallized in a mixture consisting of sodium citrate 0.86 M and Hepes 0.1 M pH 8.4. Crystals were flash-cooled in liquid nitrogen after brief soaking in a cryoprotectant consisting of 80% of reservoir added with 20% glycerol.

### Characterisation of mAb R31C2

R31C2, a monoclonal antibody raised in an A0 rat against the W1 *P*. *knowlesi* strain [6], belongs to the IgG2a isotype. R31C2 was sequenced from total RNA extracted from a hybridoma cell pellet (Fusion Antibodies Ltd, Belfast, Northern Ireland). cDNA was produced from the RNA by reverse transcription with an oligo(dT) primer. Both amplified heavy-chain variable domain (V_H_) and light-chain variable domain (V_L_) PCR products using variable domain primers were cloned into the Invitrogen sequencing vector pCR2.1 and transformed into TOP10 cells. Positive clones were identified by colony PCR. The sequences have been deposited in GenBank with entry number KM225619 for V_L_ and KM225620 for V_H_.

### Preparation of Fab R31C2 and its complex with PkAMA1

The Fab fragment of R31C2 was obtained by papain digestion and purified on a DEAE-Sephacel support in batch, then on a mono Q column to resolve different crystallizable isoforms. The complex was obtained by 1 h incubation in a 1:1 molar ratio of the recombinant protein with the Fab fragment of R31C2. A volume of 1 μl of the complex was mixed with 1 μl of buffer containing 8% PEG 6000, 40 mM Hepes pH7 and 80 mM NaCl. The drop was set up over a reservoir of 500 μl of buffer and stored at 17°C. Crystals were flash-cooled in liquid nitrogen after brief soaking in a cryoprotectant consisting of 20% PEG 6000, 50 mM Hepes pH 7, 100 mM sodium chloride and 20% glycerol.

### Diffraction data collection and structure determination

X-ray diffraction data for PkAMA1 were collected on beamline BM14 at the ESRF (Grenoble, France), and for the PkAMA1-Fab R31C2 complex on beamline PROXIMA-1 at synchrotron SOLEIL (St Aubin, France). The diffraction images were integrated with the program XDS [41] and crystallographic calculations were carried out with programs from the CCP4 program suite [42]. The PkAMA1 structure was solved by molecular replacement with PHASER software [43] using PvAMA1 (PDB code 1W81) as template. The PvAMA1 coordinates were also used as the antigen template in the R31C2 complex. The V_H_ coordinates from PDB entry 2GCY and the V_L_ coordinates from 1D5I, which showed the best sequence identity with the respective domains of R31C2, were used for the R31C2 variable dimer, and the C_H_ and C_L_ domains from PDB entry 1IGF were used as the search model for the C dimer. Two independent protein molecules were identified in the crystallographic asymmetric unit for PkAMA1 and a single complex for PkAMA1-Fab R31C2.

The structures were refined with the program BUSTER [44] and manual adjustments were made to the models with Coot [45]. The crystals parameters, data statistics and final refinement parameters are shown in Table 1 and Table 2. All structural figures were generated with PyMOL (http://www.pymol.org) [46]. The structures were deposited in the Protein Data Bank under the accession codes 4UV6 (PkAMA1) and 4UAO (PkAMA1-Fab R31C2).

**Table 1:** Crystallographic data.

	PkAMA1	PkAMA1-FabR31C2
Spacegroup	C2	C2
a, b, c (Å)	90.32, 105.70, 104.75	165.86, 71.81, 90.69
β (deg.)	98.04	116.37
Z	4	2
V_M_ (A^3^ Da^-1^)	2.9	2.7
Resolution (Å)	47.09–2.45 (2.58–2.45)	43.35–3.10 (3.27–3.10)
Unique reflections	34848 (4876)	17127 (2506)
Redundancy	2.9 (2.5)	3.1 (3.2)
Completeness (%)	97.3 (93.3)	97.8 (99.1)
Rmerge	0.100 (0.499)	0.152 (0.778)
Rpim	0.069 (0.376)	0.097 (0.497)
I/σ(I)	9.7 (1.9)	7.8 (1.3)
CC(1/2)*	0.99 (0.59)	0.99 (0.70)

* Pearson correlation coefficient of two half data sets [58].

**Table 2:** Refinement statistics.

	PkAMA1	PkAMA1-FabR31C2
Resolution (Å)	47.09–2.45	40.77–3.10
R/R_*free*_	0.17/0.23	0.19/0.27
Number of atoms		
protein	5407	5988
solvent	387	-
Wilson Plot B-value (Å^2^)	42.5	71.3
Refined Baverage (Å^2^)	Mol A 39.0	PkAMA1 74.9
	Mol B 46.2	FabR31C2 69.0
RMS deviation from ideal		
bond length (Å)	0.010	0.010
bond angle (°)	1.15	1.31
Ramachandran plot		
most favoured regions (%)	95.1	86.8
allowed regions (%)	3.8	9.5

## Results

### Structure of PkAMA1

**Fig 1:**
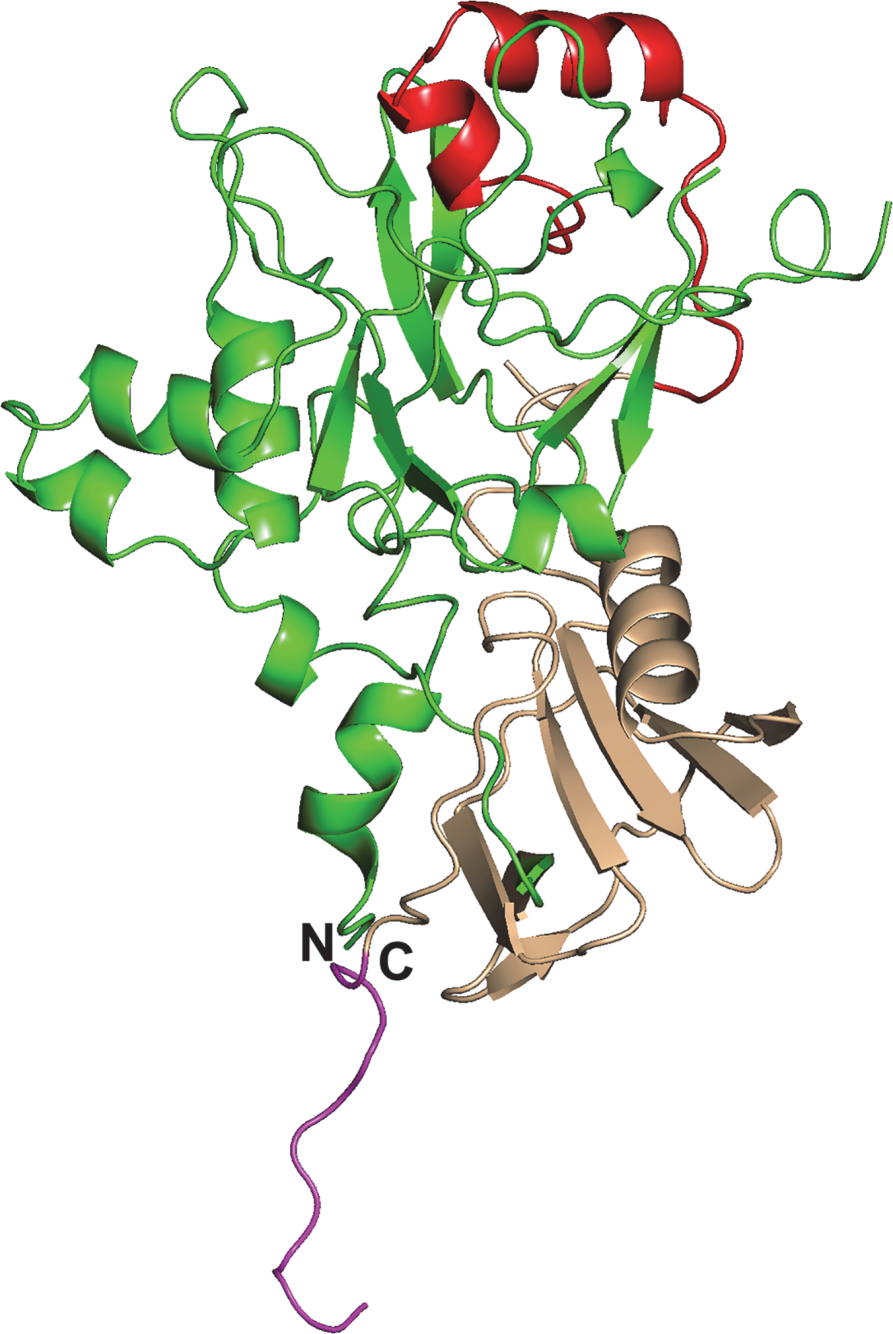
Structure of domains 1 and 2 of PkAMA1 in the non-complexed form. The structure of PkAMA1 (molecule B) is shown in ribbon representation with Domain 1 in green and Domain 2 in light brown. The Domain 2 loop is shown in red and the c-myc tail is shown in mauve. The N- and C-termini are indicated by N and C, respectively. A gap occurs within the D2 loop since the protein could not be traced from residues Gly328 to Ser332.

**Fig 2:**
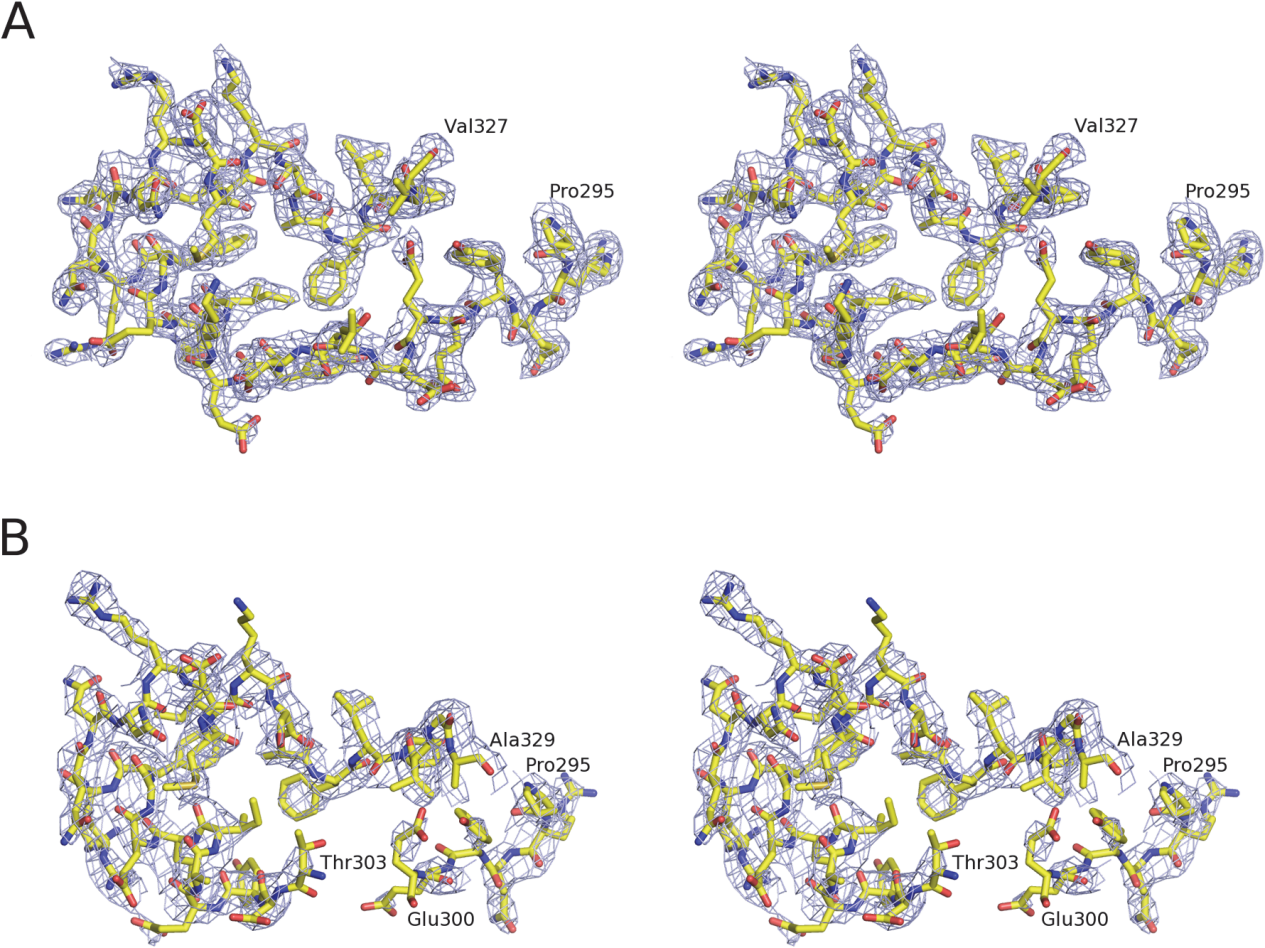
Electron density of the D2 loop. The D2 loop is shown in stereo. (A) free PkAMA1 (molecule A). (B) PkAMA1-Fab R31C2 complex. The contour level is drawn at the r.m.s. of the weighted Fobs electron density.

The free PkAMA1 protein crystallized in space group C2 with two molecules (A and B) in the asymmetric unit and the structure was refined at 2.45 Å resolution (Table 1, Table 2). The polypeptide chain of molecule A was traced from Ser52 to Glu397 (thus including eight residues of the c-myc tag), with main-chain gaps Ala131-Asp132, Ser212-Lys215 and Gly328-Asn331 due to conformational mobility. Molecule B (Fig 1) was built from Ser52 to Asp398 with main-chain gaps Ser212-Ala217 and Gly328-Ser332. The two molecules are very similar, with an r.m.s.d. of 0.34 Å in Cα positions over the 332 common residues in the PkAMA1 sequence. The D2 loop of PkAMA1, which is displaced upon binding of the RON2 component of the RON receptor complex in the homologues TgAMA1 and PfAMA1 [27,28], could be completely traced in both molecules except for the short region Gly328-Ser332 (Fig 2A). Nonetheless, the high temperature factors of this region indicate a propensity to structural mobility.

### Structure of the PkAMA1-Fab R31C2 complex

**Fig 3:**
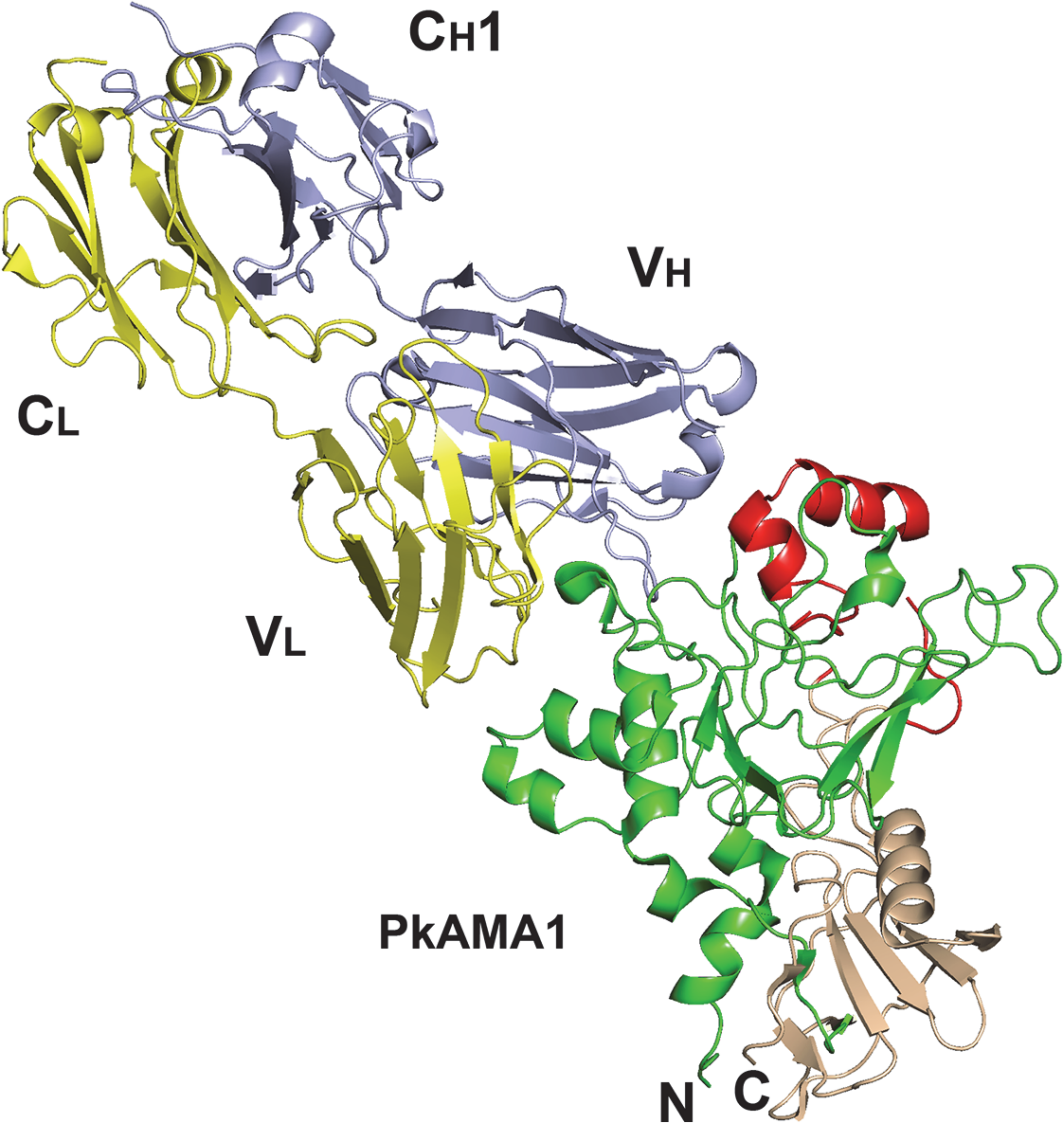
Structure of the PkAMA1-Fab-R31C2 complex. The structure of the complex is shown in ribbon representation. Domain 1 of PkAMA1 is shown in green and Domain 2 in light brown, with the Domain 2 loop in red. The light chain of Fab-R31C2 is shown in yellow (variable and constant domains labeled V_L_ and C_L_, respectively) and the heavy chain is shown in light blue (variable and constant domains labeled V_H_ and C_H_1, respectively).

**Fig 4:**
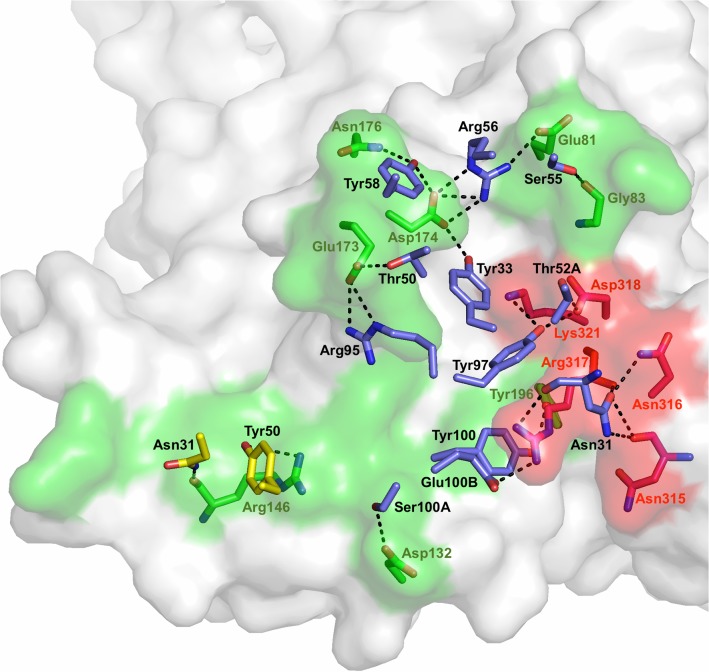
The PkAMA1 epitope recognized by R31C2. PkAMA1 is shown in surface representation with the epitope residues on Domain 1 in green and the epitope residues on the D2 loop in red. In addition, residues of both PkAMA1 and R31C2 that are involved in polar interactions are drawn in stick representation with hydrogen bonds indicated by dotted lines. The stick models of the PkAMA1 residues and their labels are green for Domain 1 and red for the D2 loop. Stick models for R31C2 are blue for V_H_ residues and yellow for V_L_ residues with residue labels in black. (The complete list of PkAMA1 and R31C2 residues in contact is given in Table 3, Table 4).

The PkAMA1-Fab R31C2 complex, which crystallized in the space group C2 with one molecule of complex in the asymmetric unit, was refined at 3.1 Å resolution (Table 1, Table 2). The main chain of PkAMA1 was traced from Met51 to Phe386, with gaps Glu301-Met302 and Phe330-Asn331 which occur in the D2 loop (Fig 2B). The heavy and light chains of the Fab fragment were both traced in their entirety to the C-terminal cysteine residues that form the interchain disulfide bridge. (Fig 3) The Fab fragment binds to the hydrophobic groove of PkAMA1 and makes significant contact with the D2 loop. A total of 2465 Å^2^ of solvent-accessible surface is buried at the antibody-antigen interface (1180 Å^2^ from the antibody; 1285 Å^2^ from the antigen). The heavy-chain variable domain (V_H_) contributes 838 Å^2^ to the buried surface while the light-chain variable domain (V_L_) contributes 342 Å^2^. All three Complementarity-Determining Regions (CDR) of both V_H_ and V_L_ make direct contacts with PkAMA1. The shape complementarity factor (shape correlation statistic, Sc) is 0.74, which is significantly above values typical of antibody-antigen complexes [47]. The more significant participation of V_H_ is also reflected in the number and nature of the contacts across the interface; for V_H_ there are 116 interatomic distances less than 3.8 Å, of which 22 are polar (including three salt bridges) while for V_L_ there are only 25 contacts, of which two are polar (Table 3, Table 4, Fig 4). The D2 loop is contacted by CDR-H1, CDR-H2 and CDR-H3 (first, second and third CDR of V_H_, respectively), with 46 interatomic contacts < 3.8 Å.

**Table 3:** PkAMA1 residues forming the epitope recognized by R31C2.

PkAMA1 residue	No. contacts V_H_	No. contacts V_L_
Glu81	5 (1)	
Gly83	6 (1)	
Asn84	5	
Phe128	4	
Asp132	1 (1)	
Ile135	1	
Arg146		10 (2)
Tyr147		1
Glu149		5
Phe169	6	
Ile171	5	
Ala172		6
Glu173	15 (3)	3
Asp174	14 (5)	
Asn176)	1 (1)	
Thr177	2	
Tyr196	5 (1)	
Asn315	3 (2)	
Asn316	4 (1)	
Arg317	25 (3)	
Asp318	9 (2)	
Lys321	5 (1)	

Column 1: residue in contact with the antibody; column 2: total number of interatomic contacts < 3.8 Å made by the residue with V_H_; column 3 total number of contacts of residue with V_L_. The number of polar contacts is given in parentheses.

**Table 4:** R31C2 residues contacting PkAMA1.

CDR	Residue	No. contacts
CDR-H1	Ser30	1
	Asn31	16 (5)
	Tyr32	4
	Tyr33	2 (1)
FW-H2	Trp47	2
CDR-H2	Thr50	3 (1)
	Thr52A	3 (1)
	Ser53	7
	Ser55	5 (1)
	Arg56	14 (4)
	Tyr58	12 (2)
CDR-H3	Arg95	4 (2)
	Tyr97	20 (2)
	Gly98	2
	Gly99	10
	Tyr100	5 (1)
	Ser100A	1 (1)
	Glu100B	5 (1)
CDR-L1	Asn31	1 (1)
CDR-L2	Tyr50	10 (1)
	Arg66	3
	Ser67	2
CDR-L3	Tyr91	2
	Lys92	3
	Gln93	1
	Leu96	3

Column 1: the Complementarity-Determining Region (CDR) or Framework region (FW) of the heavy or light chain to which the residue belongs; column 2: residue in contact with PkAMA1; column 3: total number of interatomic distances < 3.8 Å between the antibody residue and PkAMA1, with the number of polar contacts given in parentheses.

Conformational changes that occur in PkAMA1 upon binding R31C2 are restricted mainly to flexible loops that come into contact with the antibody. The largest changes are induced in the PkAMA1 loop Phe169-Asn178, where differences exceed 7 Å at its extremity. A single turn of α-helix is formed from residues Ala172 to Gln175, placing Glu173 and Asp174 in position to form a salt bridge with R31C2 heavy chain residues Arg-H95 and Arg-H56, respectively. This region of PkAMA1 makes the most extensive interactions with R31C2, with residues from CDR-H1, CDR-H2, CDR-H3 and CDR-L1 participating in direct contacts with the antigen. The loop 128–136 becomes more ordered in comparison to the unbound PkAMA1 structure with differences of up to 6 Å between the unbound and bound states. Although the D2 loop makes extensive interactions with R31C2, the regions in direct contact show only small differences. The largest of these differences occur close to the N- and C-termini of the loop, which are very mobile in both PkAMA1 structures (as well as in PfAMA1 and PvAMA1) and do not contact the antibody. Most intramolecular contacts of the D2 loop are with Domain 1 of the PkAMA1 core; Glu288 is the only Domain 2 residue to contact the D2 loop but this is with Arg296 at its N-terminus.

### Consequences of somatic mutations in R31C2 on PkAMA1 binding

**Fig 5:**
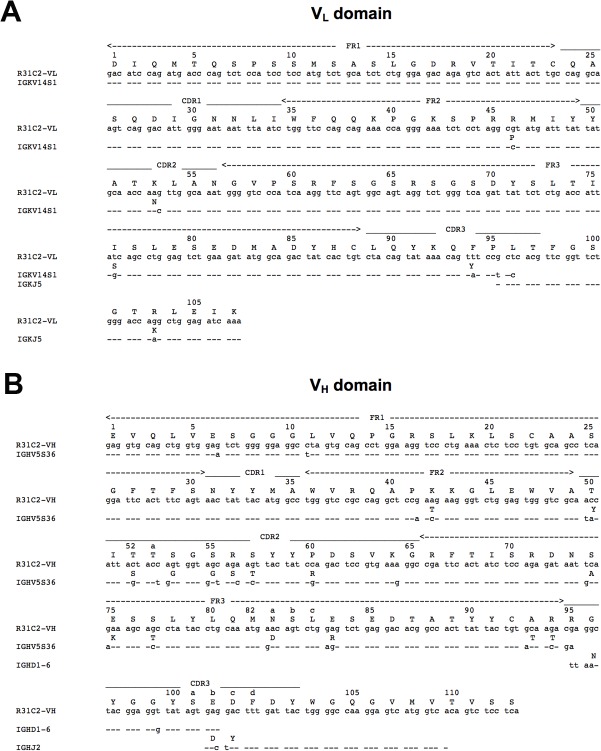
Germline sequences and somatic mutations of the R31C2 variable domains. (A) Amino acid and nucleotide sequences of the R31C2 V_L_ domain compared with the *Vκ* and *Jκ* germline genes. (B) Amino acid and nucleotide sequences of the R31C2 V_H_ domain compared with the *VH*, *D* and *JH* germline genes.

The V_H_ and V_L_ sequences were compared with germline sequences using the International Immunogenetics Information server [48] (Fig 5). The V_L_ domain derives from the Vκ germline gene IGKV14S1 (98.9% identity) and the J gene segment IGKJ5. Five somatic mutations in the amino acid sequence are present in the regions encoded by the Vκ and Jκ gene segments but none of these make direct contacts with PkAMA1. The V_H_ domain derives from the germline gene IGHV5S36 (92.7% identity), the IGHD1-6 D minigene and the IGHJ2 J gene segment. Eighteen somatic amino acid mutations are present, five of which contact PkAMA1: Tyr50Thr, Gly53Ser, Gly55Ser and Ser56Arg in CDR-H2 and Asp100bGlu in CDR-H3. The higher selection pressure on V_H_ probably reflects the significantly more important role of this domain in binding to PkAMA1. The somatic mutation Tyr50Thr leads to a hydrogen bond between Oγ and the glutamate group of Glu173, which would not be sterically possible for the germline residue tyrosine. The mutation Gly55Ser does not lead to additional polar interactions but probably improves the complementarity at the antibody-antigen interface. The mutation Ser56Arg appears to have the most significant impact in the affinity maturation of R31C2 as this antibody residue forms a salt bridge to Glu81 and Asp174 of PkAMA1. CDR-H3 residue Arg95, which forms a salt bridge to Glu173, probably results from nucleotide deletions and additions occurring at the junction between V_H_ and D (used in the third reading frame).

## Discussion

R31C2 was the first invasion-inhibitory anti-AMA1 mAb to be characterized, providing the first demonstration that AMA1 plays a crucial role in the infection of RBCs by *Plasmodium* merozoites and highlighting the potential of this parasite protein as a malaria vaccine candidate [6]. *P*. *knowlesi* merozoites are able to attach to the RBC surface in the presence of R31C2 but no penetration of the host cell is observed, implying that AMA1 comes into play after initial contact by the parasite [35]. The accumulated results of many studies with *Plasmodium* and other *Apicomplexa* parasites have since confirmed this view. AMA1 has been shown to bind to RON2, a component of the parasite RON protein complex that is transferred to the RBC membrane during invasion [25, 27, 28]. This interaction is thought to occur at the tight junction formed between the invading merozoite and the RBC.

**Fig 6:**
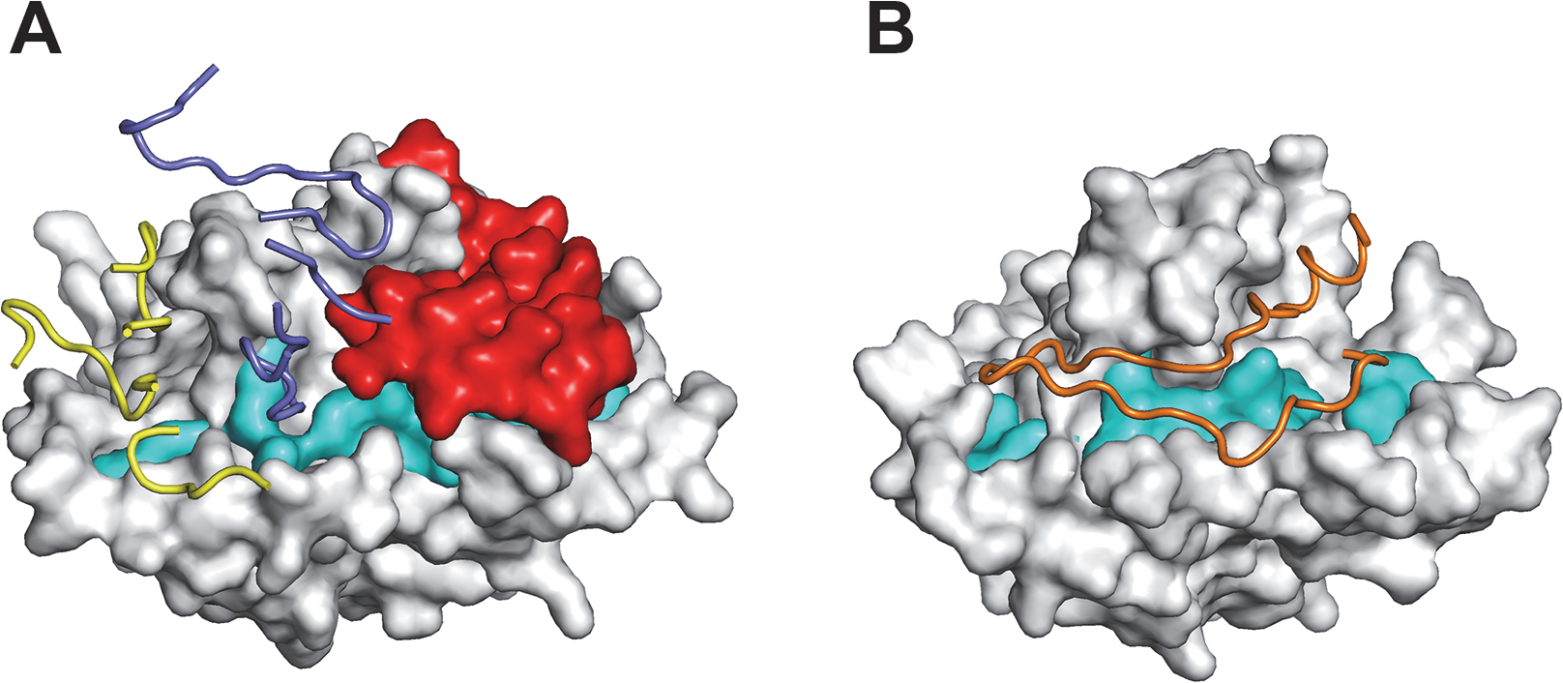
Mechanism of receptor-binding inhibition by monoclonal antibody R31C2. (A) R31C2 blocks interaction of the RON2 receptor by occupying the hydrophobic groove and preventing movement of the D2 loop. PkAMA1 is shown in surface representation with Domain 1 residues lining the hydrophobic groove that are invariant or well conserved across species [18] shown in cyan and the D2 loop shown in red. The CDR residues of R31C2 are shown in ribbon representation; those of V_H_ are blue and those of V_L_ are yellow. (B) Structure of PfAMA1 complexed with the PfRON2 peptide (PDB entry 3ZWZ) [28]. PfAMA1 is shown in surface representation and in the same orientation as PkAMA1 in (A). Species-conserved residues of Domain 1 that line the hydrophobic groove are shown in cyan. The displaced D2 loop is not visible in this structure, probably due to high mobility. Binding of the PfRON2 peptide, shown here in orange as ribbon representation, requires displacement of the D2 loop to expose the complete receptor-bing site.

The RON2-binding site on AMA1 comprises the hydrophobic groove and an adjacent region that becomes exposed after displacement of the D2 loop by the receptor [27, 28]. In the crystal structure of the PkAMA1-Fab R31C2 complex, the antibody binds not only to the hydrophobic groove but also to the D2 loop (Fig 6A). The D2 loop makes significant contacts with R31C2 (52 out of 142 interatomic contacts <3.8 Å, including nine hydrogen bonds); it nonetheless preserves the same conformation as found in the unbound PkAMA1. The RON2-binding site is thus inaccessible to the receptor in the presence of R31C2 because interactions with the mAb keep the D2 loop firmly in place as well as blocking access to a large fraction of the hydrophobic groove (Fig 6B). Structural analyses of two other complexes of AMA1 with invasion-inhibitory anti-PfAMA1 mAbs, 1F9 [21] and the IgNAR 14I [22], have shown that these also bind to the hydrophobic groove but their epitopes are displaced towards one end of the groove and do not include the D2 loop. By contrast, epitope mapping of the anti-PfAMA1 mAb 4G2, another extensively studied invasion-inhibitory antibody, shows that this antibody does not bind to the hydrophobic groove but rather to the base of the D2 loop [17, 49]. The mechanism of inhibition in this case is thus most likely indirect by preventing displacement of the D2 loop to fully expose the RON2-binding site. By targeting both the hydrophobic groove and the D2 loop, R31C2 is thus a very effective inhibitor of RBC invasion.

**Fig 7:**
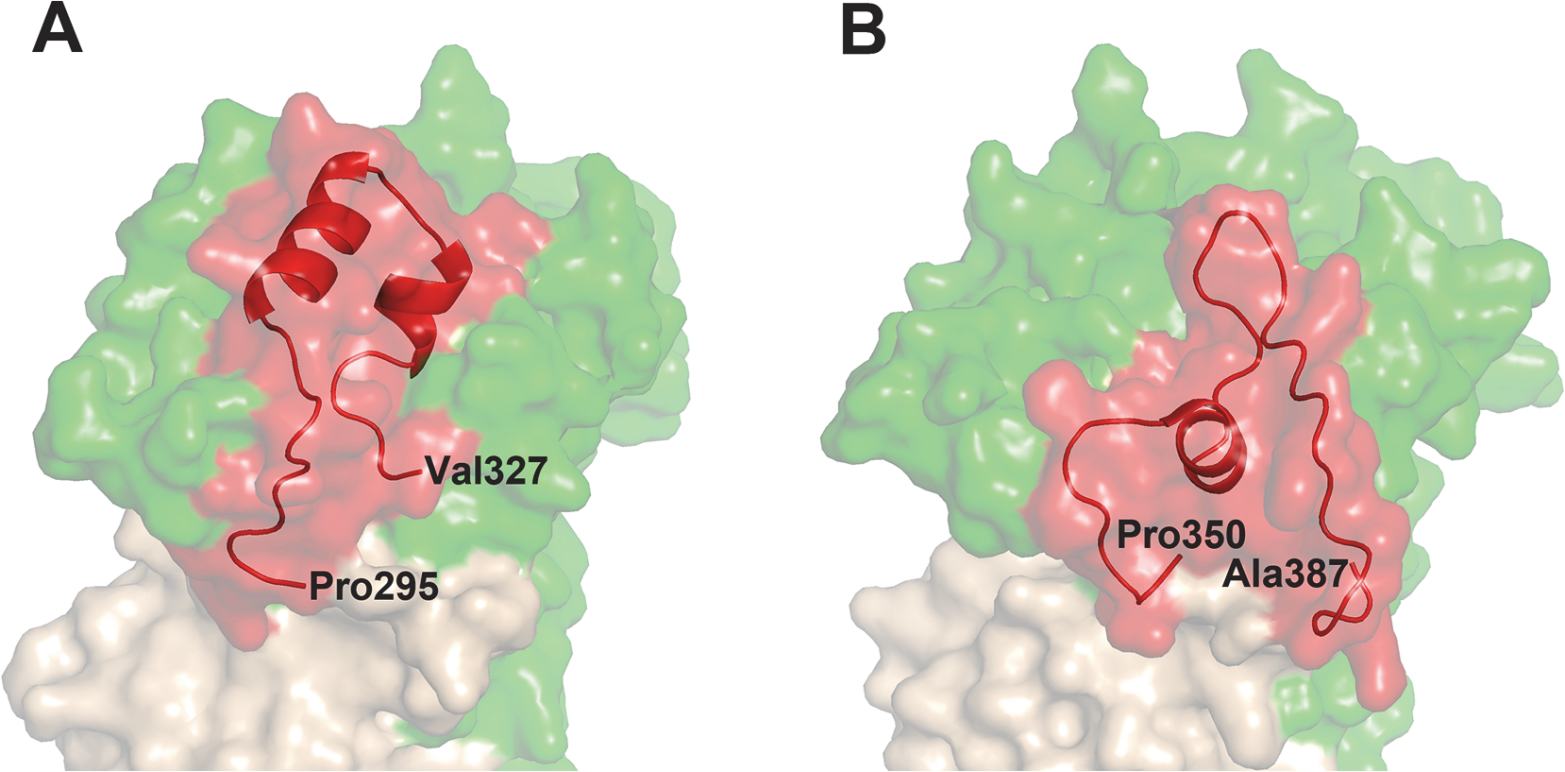
Comparison of the D2 loop of PkAMA1 and PfAMA1. (A) PkAMA1. (B) PfAMA1 (PDB entry 2Z8V). The two structures are viewed from equivalent orientations and are shown in surface representation with Domain 1 in green, Domain 2 in light brown, and the D2 loop in red. The D2 loop conformation of both orthologues is shown in ribbon representation and the N- and C-terminal residues are labelled.

The D2 loop is functionally important as it must be displaced to fully expose the binding site for the RON receptor. Nonetheless, this region is more variable among the different *Plasmodium* homologues than is the sequence variability over the complete ectodomain [50]. Interestingly, the D2 loop adopts a different conformation in PkAMA1 to that which has been observed in PfAMA1 (Fig 7). This could be due to sequence differences between the two homologues or could arise from inherent flexibility in this region of AMA1.

Polymorphism in AMA1 has been studied most extensively in *P*. *falciparum* [51, 52]. In PfAMA1, polymorphism has most likely occurred in response to the host’s immune defenses since non-synonymous differences in the nucleotide sequences predominate over synonymous differences. PvAMA1 is also highly polymorphic [50, 53–56]. We have studied polymorphism in PkAMA1 to understand its effects on protein structure and function, and on the immune response of the host [57]. A total of 21 polymorphic residues were detected in the 52 isolates examined: four in Domain 1, seven in Domain 2 and five in Domain 3. The pattern of polymorphism thus differs from that found in PfAMA1 and PvAMA1, since 14 of the polymorphic sites in PkAMA1 align with sites that are non-polymorphic in the two other homologues. No polymorphisms were located around the periphery of the RON2-binding site of PkAMA1, in contrast to PfAMA1 and PvAMA1 where variability in these regions has been suggested to play an important role in immune escape from the host response [18]. Moreover, none of the residues comprising the epitope of R31C2 are polymorphic and the mAb should thus be largely cross-strain reactive. Of note, in the high frequency polymorphism Arg296Ser, the arginine residue forms a salt bridge to Glu288; these two residues correspond to Lys351 and Glu343 in PfAMA1, which do not form a salt bridge [18]. It is unclear if this PkAMA1 polymorphism influences the D2 loop conformation. Nonetheless, the absence of polymorphism close to the receptor-binding site of PkAMA1 suggests that, unlike PfAMA1 and PvAMA1, a single variant sequence may be sufficient for an effective vaccine formulation against *P*. *knowlesi* malaria.
